# Marked Parathyroid Hormone (PTH)-Independent Hypercalcemia Revealing Sarcoidosis Mimicking Malignancy: A Case Report

**DOI:** 10.7759/cureus.113873

**Published:** 2026-08-03

**Authors:** Maneesh Kumar Reddy Gopireddy, Kaandeeban Mohanraj, John Amerson, Naveen Punchayil Narayanankutty

**Affiliations:** 1 Internal Medicine, Narayana Medical College and Hospital, Nellore, IND; 2 Internal Medicine, Indira Gandhi Medical College and Research Institute, Puducherry, IND; 3 Nephrology, University of South Florida, Tampa, USA

**Keywords:** acute kidney injury, hypercalcemia, nephrolithiasis, pth-independent hypercalcemia, sarcoidosis

## Abstract

Severe hypercalcemia is a life-threatening emergency and requires prompt management. Sarcoidosis is a multisystem granulomatous disease that often involves the lungs but may also involve other systems, leading to extrapulmonary manifestations. The presence of lymphadenopathy, constitutional symptoms, and renal dysfunction can mimic that of malignancy, giving rise to diagnostic uncertainty in some cases.

Here we present a 57-year-old male who came with complaints of fatigue, nausea, and right flank pain. Laboratory investigations revealed clinically significant hypercalcemia (13.8 mg/dL) with acute kidney injury (blood urea nitrogen (BUN) 26 mg/dL, serum Cr 2.9 mg/dL) and normal parathyroid hormone (PTH), 30.1 pg/mL. The initial imaging raised the suspicion of malignancy due to the presence of diffuse intra-abdominal and mediastinal lymphadenopathy and splenomegaly. Additionally, there were elevated 1,25-dihydroxyvitamin D and angiotensin-converting enzyme levels with normal PTH-related peptide levels. Bone marrow biopsy gave the picture of extensive non-necrotizing granulomatous inflammation without evidence of lymphoma or leukemia, narrowing the diagnosis towards sarcoidosis. Following treatment with intravenous hydration, zoledronic acid, and subsequent corticosteroid therapy after exclusion of alternative causes of hypercalcemia, calcium levels and renal function returned to baseline.

This case highlights the need to consider sarcoidosis as a differential diagnosis in patients with hypercalcemia and normal PTH levels. To avoid misdiagnosis and to allow prompt treatment, a structured diagnostic method with timely histopathological confirmation is needed.

## Introduction

Sarcoidosis is a difficult disease to recognize because it can affect many organs and often does not follow a typical pattern. With an annual incidence of 17 to 35 per 100000 population, it usually involves the lungs and lymphatics, but its manifestations could stretch beyond to other organ systems such as skin, eyes, heart, and kidneys [1-3].

About 6% of people with sarcoidosis present with hypercalcemia. This elevated calcium level usually drops the parathyroid hormone (PTH), but normal values are also reported. Additionally, 42% of the untreated cases result in progression to renal failure [4,5]. The culprit in focus associated with raised calcium levels is the increased activity of 1α-hydroxylase, leading to the elevated extrarenal production of 1,25-dihydroxyvitamin D [6,7].

A level of total calcium of 14 mg/dL or greater (> 3.5 mmol/L) or ionized calcium of 10 mg/dL or greater (≥ 2.5 mmol/L) can be classified as a medical emergency that requires prompt management. Approximately 90% of cases of hypercalcemia are attributable to primary hyperparathyroidism or malignancy [4,5].

In clinical practice, sarcoidosis is often considered a diagnosis of exclusion; however, a definitive diagnosis requires a compatible clinical presentation, histopathologic evidence of non-necrotizing granulomatous inflammation, and exclusion of alternative causes. Therefore, patients presenting with hypercalcemia, constitutional symptoms, diffuse lymphadenopathy, and renal impairment require a systematic evaluation to distinguish sarcoidosis from other granulomatous, infectious, and malignant conditions with similar clinical presentations [5,8].

In this report, we describe a patient who presented with marked PTH-independent hypercalcemia, acute kidney injury, recurrent kidney stones, and diffuse lymphadenopathy, initially raising concern for an underlying malignancy. This case highlights how hypercalcemia differentials cloud the diagnoses and underestimate the importance of considering it in patients with unexplained hypercalcemia and renal involvement to allow timely, targeted treatment.

## Case presentation

A 57-year-old male with a medical history of chronic obstructive pulmonary disease, hypertension, obesity, and immune thrombocytopenic purpura arrived at the hospital’s emergency department with complaints of fatigue, nausea, and right flank pain for the past week. He had no history of fever, chills, hematuria, dysuria, chest pain, edema, dyspnea, recent sick contacts, or use of over-the-counter medications.

He was previously hospitalized for bilateral renal calculi, hypercalcemia, and diffuse lymphadenopathy. He received intravenous fluids and calcitonin for hypercalcemia. A right nephrostomy tube was placed for obstructive nephrolithiasis, followed by lithotripsy, and the nephrostomy tube was removed one week later. A lymph node biopsy was performed, but it was nondiagnostic because of inadequate tissue sampling.

He has a significant smoking history of half a pack of cigarettes a day for 20 years. No other significant family history. During the current admission, his vitals were blood pressure 153/72 mm/Hg, pulse rate 60 bpm, respiratory rate 19 breaths/min, temperature 98.6°F (37°C), and SpO₂ 95% on room air, and the rest of the examination was normal. Initial laboratory investigations revealed hypercalcemia, with derangements in renal parameters. The PTH was not appropriately elevated in the setting of hypercalcemia, ruling out hyperparathyroidism to push the cause as a PTH-independent process.

Urinalysis results were consistent with microscopic hematuria and pyuria. Twenty-four-hour urinary calcium excretion was performed, showing a markedly elevated level of 854 mg/24 h, making the diagnosis of familial hypocalciuric hypercalcemia unlikely. PTH-related peptide levels were within normal limits (12 pg/ml). PTHrP was measured as part of the evaluation of PTH-independent hypercalcemia to exclude humoral hypercalcemia of malignancy. Initial laboratory investigations are summarized in Table 1.

**Table 1:** Key laboratory findings at the time of initial diagnosis

Laboratory Parameter	Value	Reference Range
Serum Calcium	13.8 mg/dL	8.6–10.2 mg/dL
Ionized Calcium	1.69 mmol/L	1.12–1.32 mmol/L
Creatinine	2.9 mg/dL	0.7–1.3 mg/dL
Blood Urea Nitrogen (BUN)	26 mg/dL	7–20 mg/dL
Estimated Glomerular Filtration Rate (eGFR)	24 mL/min/1.73 m²	>60 mL/min/1.73 m²
Parathyroid Hormone (PTH)	30.1 pg/mL	15–65 pg/mL
Serum 25-Hydroxyvitamin D	10.7 ng/mL	30–100 ng/mL
Serum Uric Acid	11.3 mg/dL	3.5–7.2 mg/dL
White Blood Cell Count (WBC)	4.15 ×10³/µL	4.0–11.0 ×10³/µL
Hemoglobin (Hgb)	11.4 g/dL	13.5–17.5 g/dL
Thyroid-Stimulating Hormone (TSH)	1.53 µIU/mL	0.4–4.0 µIU/mL
Urine Appearance	Cloudy	Clear
Urine Leukocyte Esterase	Trace	Negative
Urine Hemoglobin	Moderate	Negative
Urine Protein	30 mg/dL	Negative
Urine White Blood Cells (WBC)	21–50 /HPF	0–5 /HPF
Urine Red Blood Cells (RBC)	51–100 /HPF	0–2 /HPF

**Figure 1:**
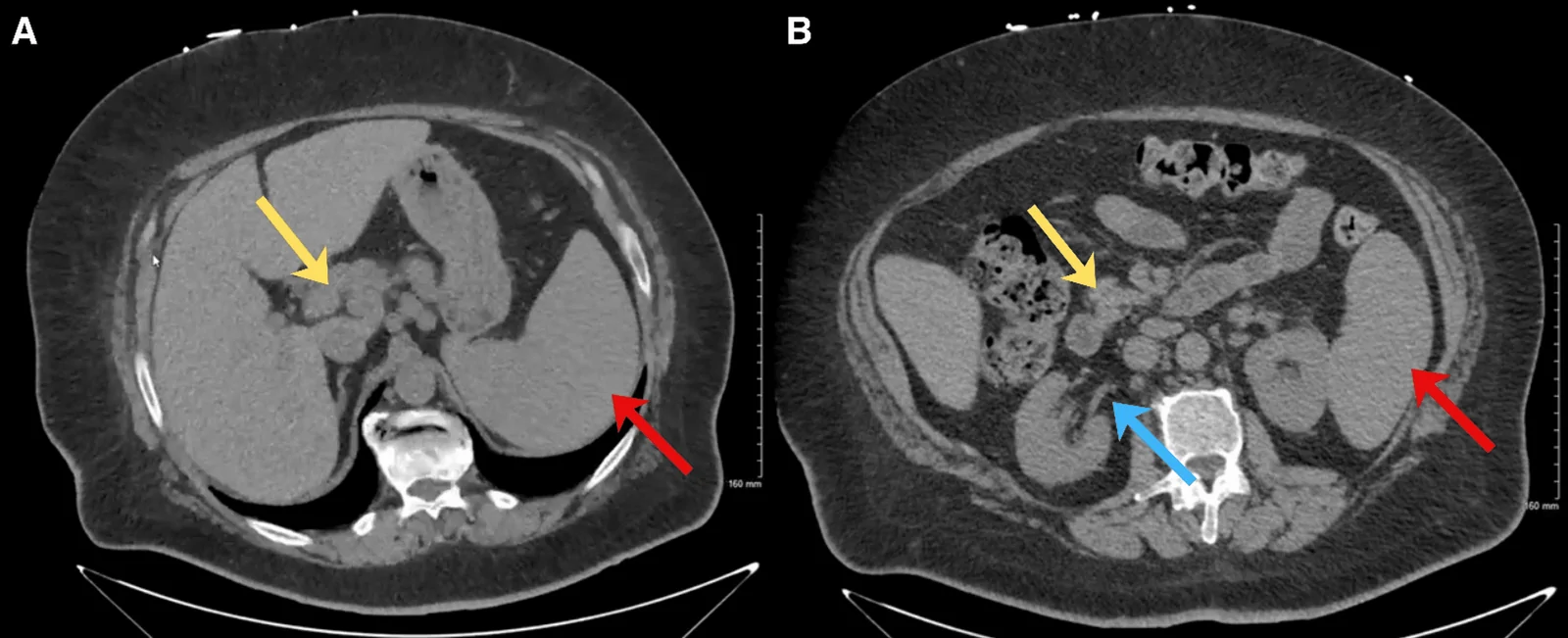
Computed tomography of the abdomen and pelvis demonstrating systemic lymphadenopathy, splenomegaly, and mild hydronephrosis A: splenomegaly (red arrow) and upper abdominal lymphadenopathy (yellow arrow); B: splenomegaly (red arrow), retroperitoneal lymphadenopathy (yellow arrow), and mild right hydronephrosis (blue arrow).

Computed tomography of the abdomen and pelvis demonstrated splenomegaly and upper abdominal lymphadenopathy (Figure 1A) and retroperitoneal lymphadenopathy with mild right hydronephrosis (Figure 1B).

**Figure 2:**
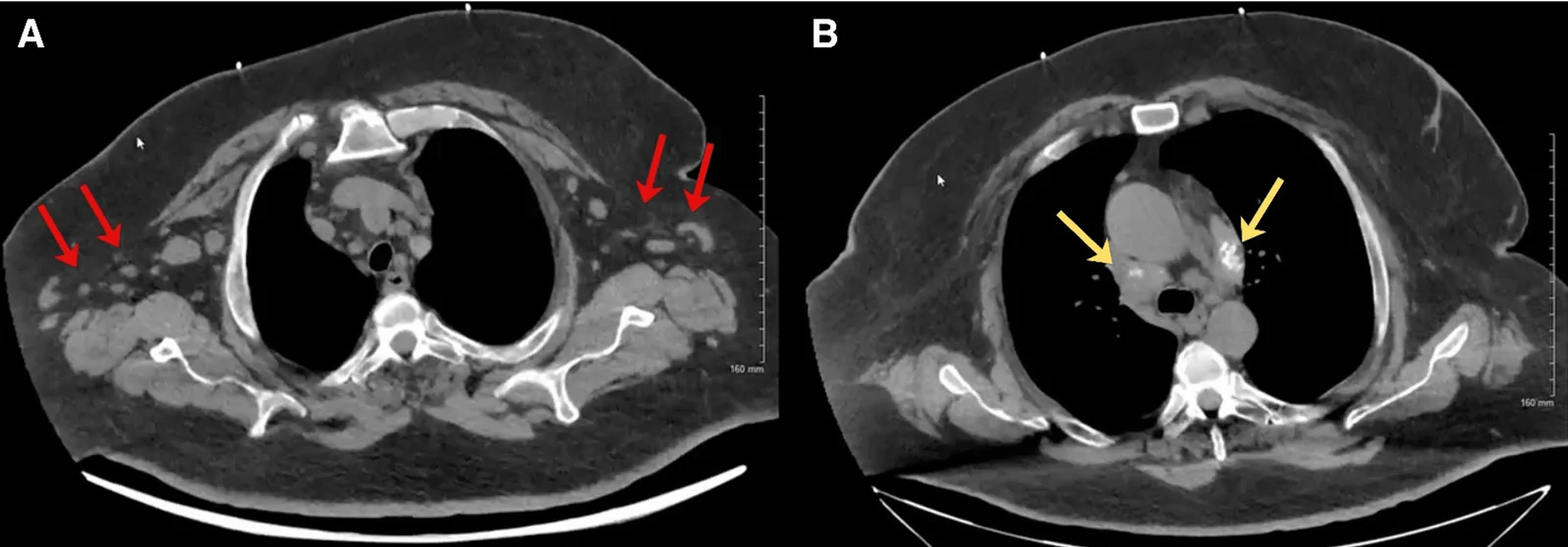
Computed tomography of the chest demonstrating bilateral axillary and mediastinal lymphadenopathy A: bilateral enlarged axillary lymph nodes (red arrows); B: enlarged calcified mediastinal and hilar lymph nodes (yellow arrows).

Computed tomography of the chest demonstrated bilateral enlarged axillary lymph nodes (Figure 2A) and calcified mediastinal and hilar lymphadenopathy (Figure 2B).

Finally, multiple small bilateral cervical lymph nodes were seen on computed tomography of the neck. None of the lymph nodes appeared suspicious enough to require further examination.

Later, a technetium-99m sestamibi parathyroid scan was performed to exclude occult primary hyperparathyroidism and showed no evidence of active focal parathyroid adenoma. Further evaluation in view of the cause of PTH-independent hypercalcemia demonstrated elevated serum 1,25-dihydroxyvitamin D levels at 111 pg/mL and elevated angiotensin-converting enzyme levels at 163.3 U/L.

These findings increased suspicion for a granulomatous disease, while lymphoma and infectious granulomatous diseases remained important differential diagnoses. A bone marrow biopsy was subsequently performed since the lymph node biopsy from the previous admission was inconclusive regarding any diagnosis.

Normocellular marrow with preserved trilineage hematopoiesis and extensive non-necrotizing granulomatous inflammation; additionally, no evidence of lymphoma or leukemia was seen on histopathological examination. After exclusion of malignancy and infectious granulomatous diseases based on the available clinical evaluation, the overall clinicopathologic findings were considered consistent with sarcoidosis. Table 2 summarizes the important facts and diagnostic rationale that either support or contradict each diagnosis.

**Table 2:** Differential diagnoses considered during the evaluation of marked PTH-independent hypercalcemia PTH: parathyroid hormone

Diagnosis	Supporting Findings	Findings Against
Lymphoma	Diffuse lymphadenopathy, splenomegaly	Lymph node and bone marrow biopsies were negative for malignancy
Primary hyperparathyroidism	Marked hypercalcemia	PTH not appropriately elevated in the setting of hypercalcemia; negative sestamibi scan
Familial hypocalciuric hypercalcemia (FHH)	Hypercalcemia	Markedly elevated 24-hour urinary calcium excretion
Sarcoidosis	Elevated ACE and 1,25-dihydroxyvitamin D levels	Histopathology ultimately confirmed the diagnosis

The patient was hence treated with intravenous 0.9% normal saline hydration at a rate of 200-300 mL/hour, zoledronic acid 4 mg intravenously, and corticosteroid therapy (prednisone 20 mg per day) during the stay in the health facility, resulting in progressive normalization of calcium levels and improvement in renal function. He was discharged following clinical improvement and was later scheduled for follow-up in two weeks' time with the nephrology clinic.

## Discussion

Sarcoidosis is a rare cause within the differential spectrum of hypercalcemia, thus posing significant diagnostic challenges. The most reported causes of elevated calcium levels are due to primary hyperparathyroidism and malignancy, which account for approximately 90% of the cases. The initial diagnostic approach to hypercalcemia is to distinguish PTH-dependent from PTH-independent causes based on serum PTH levels. The absence of stronger specific diagnostic tools to rule out these overlapping differential diagnoses leads to delays in recognition of the condition and misdiagnosis [1-4].

The pathogenesis of hypercalcemia in granulomatous diseases such as sarcoidosis is most commonly mediated by increased extrarenal production of 1,25-dihydroxyvitamin D by activated macrophages within granulomas through the enzyme 1α-hydroxylase. This is not tightly regulated by the PTH like the renal 1α-hydroxylase. Thereby leading to enhanced intestinal calcium absorption, hypercalcemia, and hypercalciuria. In our patient, elevated 1,25-dihydroxyvitamin D and calcium in the setting of normal PTH suggested a PTH-independent mechanism as the cause of hypercalcemia. The subsequent hypercalciuria likely contributed to the recurrent nephrolithiasis and renal dysfunction [6,7,9].

This case highlights a complex clinical presentation of sarcoidosis characterized by marked PTH-independent hypercalcemia, acute kidney injury, recurrent nephrolithiasis, and diffuse lymphadenopathy, initially raising concern for lymphoproliferative malignancy. The initial evaluation focused on excluding infectious granulomatous diseases and lymphoproliferative disorders based on the clinical history, laboratory findings, imaging, and histopathologic evaluation [1-3]. Thus, following a multitude of investigations and finally the histopathology of the bone marrow specimen, we have concluded that sarcoidosis is the definitive diagnosis.

A systematic approach to PTH-independent hypercalcemia was critical in this case, and it includes differential diagnoses such as malignancy, granulomatous diseases, vitamin D intoxication, medication-induced hypercalcemia, and familial hypocalciuric hypercalcemia. Parathyroid-related causes were less likely in our patient following normal PTH levels and a negative sestamibi parathyroid scan [4,8]. Additionally, the presence of significantly elevated 24-hour urinary calcium excretion effectively ruled out familial hypocalciuric hypercalcemia [10].

Malignancy-associated hypercalcemia was also considered, given the patient’s extensive lymphadenopathy, smoking history, anemia, and splenomegaly. However, normal PTH-related peptide levels, absence of malignant findings on imaging, and bone marrow biopsy results showing no evidence of lymphoma or leukemia reduced the likelihood of malignancy [4,8,11,12].

Also, a close relationship between sarcoidosis and lymphoproliferative disorders has been described. It is noted that chronic sarcoidosis and bone marrow involvement have a higher propensity to develop lymphoma in the span of two to eight years following the initial diagnosis. This entity is coined under a term called sarcoidosis-lymphoma syndrome [13,14].

Thus, distinguishing between sarcoidosis and lymphoma can be particularly challenging because both conditions may present with constitutional symptoms, generalized lymphadenopathy, splenomegaly, and elevated calcitriol levels. There are several recent reported cases that describe sarcoidosis presenting with a similar dyad of hypercalcemia and diffuse lymphadenopathy. Similar to these cases, our patient underwent an elaborate evaluation for malignancy before the final diagnosis. This highlights the need to maintain a broad differential diagnosis in these clinical presentations [11,12,15].

The elevated angiotensin-converting enzyme level in this case provided supportive evidence for sarcoidosis, although angiotensin-converting enzyme lacks sufficient specificity to serve as a similar one diagnostic test [1-3,5].

One of the noteworthy features of this case was the diagnostic role of bone marrow biopsy. The initial lymph node biopsy was nondiagnostic, underscoring the challenge of obtaining adequate tissue samples in such cases. Ultimately, bone marrow biopsy revealed extensive non-necrotizing granulomatous inflammation with preserved trilineage hematopoiesis, providing histopathologic confirmation of sarcoidosis, thus being one of the conclusive diagnostic methods to distinguish sarcoidosis from other causes [5].

Although bone marrow involvement in sarcoidosis is uncommon, it has been reported and may serve as a valuable diagnostic site when lymph node sampling is inadequate. This case highlights the importance of exploring alternative tissue sources when there is high clinical suspicion despite non-diagnostic initial investigations [16,17].

Renal involvement in sarcoidosis is often underrecognized, mainly because the disease is not primarily seen as a urological disease. Some of the predominantly reported manifestations include hypercalcemia, hypercalciuria, nephrolithiasis, granulomatous interstitial nephritis, or glomerular disease and tubular dysfunction, which were similarly seen in our patient, mainly attributed to the prolonged calcium dysregulation [4]. Early recognition of sarcoidosis-related renal manifestations is essential, as delayed diagnosis can lead to irreversible renal damage [9]. Similarly, imaging findings alone are insufficient for diagnosis, reinforcing the importance of histologic confirmation in conjunction with compatible clinical and radiologic features.

Patients with sarcoidosis need close monitoring of the calcium levels, including avoiding excessive sunlight exposure and vitamin D supplementation. The initial management of the markedly high calcium levels includes aggressive intravenous fluid therapy, bisphosphonates, loop diuretics to promote calcium excretion, and corticosteroids.

According to the latest European Respiratory Society guidelines 2021, sarcoidosis-related hypercalcemia is typically treated via corticosteroid therapy with prednisone 20 mg/day initially, later tapering it to a maintenance dose, which suppresses granulomatous inflammation and reduces extrarenal vitamin D activation [3,6]. Other second-line options include immunosuppressants such as methotrexate, hydroxychloroquine, leflunomide, azathioprine, and mycophenolate mofetil [18].

When there is no improvement with the above immunosuppressants, biological and targeted synthetic therapies such as infliximab and adalimumab have also been explored in the management of this condition [19].

Zoledronic acid was included in the treatment due to its glucocorticoid-sparing effect and to address the reported rebound hypercalcemia during tapering of the steroids [20,21]. Early initiation of treatment often leads to rapid normalization of calcium levels and improvement in renal function. Long-term follow-up is required to monitor for disease relapse, chronic organ involvement, and treatment-related adverse effects [2,18]. Thus, a structured diagnostic approach, combined with persistence in obtaining histologic confirmation, is essential to avoid misdiagnosis and ensure timely management.

## Conclusions

This case describes an atypical presentation of sarcoidosis in which marked PTH-independent hypercalcemia and acute kidney injury were the dominant features, initially raising concern for an underlying malignancy. It highlights the importance of considering sarcoidosis in the differential diagnosis of unexplained PTH-independent hypercalcemia. A systematic diagnostic approach with persistence in obtaining adequate tissue for histopathological confirmation was essential to establishing the diagnosis. Early recognition and prompt corticosteroid therapy can result in significant clinical improvement and recovery of renal function.
